# A Multicenter Retrospective Study Predicting Early Noninvasive Ventilation Failure in Patients With Acute Hypoxic Respiratory Failure

**DOI:** 10.1111/crj.70098

**Published:** 2025-06-30

**Authors:** Xiaoyi Liu, Hui Liu, Lijuan Chen, Xiangde Zheng, Hui Ran, Lili Chen, Rui Zhou, Yufeng Wang

**Affiliations:** ^1^ Department of Critical Care Medicine Dazhou Central Hospital Dazhou Sichuan China; ^2^ Ophthalmology Dazhou Central Hospital Dazhou Sichuan China; ^3^ Department of Respiratory and Critical Care Medicine Sichuan Provincial People's Hospital, University of Electronic Science and Technology of China Chengdu China; ^4^ Department of Respiratory and Critical Care Medicine Dazhu County People's Hospital Dazhou Sichuan China

**Keywords:** acute hypoxic respiratory failure, endotracheal intubation, hypoxemia, noninvasive ventilation, respiratory drive, Volume OXygenation index

## Abstract

**Background:**

Volume OXygenation (VOX) index has good efficacy in predicting the failure of high‐flow nasal cannula therapy. However, its predictive value for treatment failure in patients receiving noninvasive ventilation (NIV) remains uncertain.

**Methods:**

Patients who underwent early NIV treatment were grouped based on their 2‐h NIV VOX Youden index. The low‐risk group consisted of patients with a VOX value > 20.45 (*n* = 188), while the high‐risk group included those with a VOX value ≤ 20.45 (*n* = 200). Baseline data and arterial blood gas values were collected at 2, 12, and 24 h after NIV initiation.

**Results:**

Compared to the low‐risk group, the high‐risk group exhibited higher SOFA scores, respiratory rates, and heart rates, along with a lower oxygenation index (P/F) (all *p* < 0.05). Following NIV treatment, the low‐risk group showed a more significant increase in P/F values at 2 h, 12 h, and 24 h after NIV initiation. The low‐risk group showed a lower VT and MV (minute ventilation volume) at 2 h, 12 h, and 24 h of NIV (*p* < 0.05). Moreover, the low‐risk group had a lower intubation rate (7.98% vs. 77%, *p* < 0.05) and mortality rate (4.79% vs. 17.5%, *p* < 0.05). At 2 h of NIV, the area under the receiver operating characteristic curve for predicting NIV failure using the VOX index was 0.843 (95% CI 0.805–0.882). Using a VOX value threshold of 20.45 to predict NIV failure, the sensitivity was 69.1%, and the specificity was 94.4%. Furthermore, a VOX value ≤ 20.45 was identified as an independent risk factor for tracheal intubation and death.

**Conclusions:**

VOX index shows promise to serve as an effective evaluation index to predict early NIV efficacy in patients with AHRF; a VOX value > 20.45 after 2 h of NIV treatment can better predict improvements in hypoxia, respiratory drive, and NIV outcomes, guide early tracheal intubation in cases of NIV failure, and have a certain predictive effect on patient outcomes.

## Abstract 2

VOX index shows promise to serve as an effective evaluation index to predict early NIV efficacy in patients with AHRF, a VOX value > 20.45 after 2 h of NIV treatment can better predict improvements in hypoxia and NIV outcomes, guide early tracheal intubation in cases of NIV failure, and have a certain predictive effect on patient outcomes.

**Abbreviations**
AHRF: acute hypoxic respiratory failure
NIV: noninvasive ventilation
VOX: Volume OXygenation
HACOR: heart rate, acidosis, consciousness, oxygenation, and respiratory rate
RR: respiratory rate
HR: heart rate
P/F: arterial blood oxygen partial pressure/inhaled oxygen concentration
IPAP: inspired positive airway pressure
EPAP: expiratory positive airway pressure
VT: tidal volume
MV: minute ventilation volume
COPD: chronic obstructive pulmonary disease
HFNC: high flow nasal catheter
OR: odds ratio
CI: confidence interval
ICU: intensive care unit
AIDS: acquired immune deficiency syndrome
APACHE II: Acute Physiology and Chronic Health Evaluation II
GCS: Glasgow Coma Scale
SOFA: Sequential Organ Failure Assessment
SBP: systolic blood pressure
DBP: diastolic blood pressure
WBC: white blood cell count
PLT: platelet count
Hb: hemoglobin
PaCO_2_
: partial pressure of carbon dioxide
AUC: area under the receiver operating characteristic curve

## 1 Introduction

Noninvasive ventilation (NIV) has proved to be an effective treatment for respiratory failure, demonstrating a significant reduction in mortality, from 20.6 to 14.2% [1]. Consequently, the utilization of NIV has been steadily rising over the years [2, 3]. Despite the availability of numerous NIV guidelines [4, 5, 6, 7], the failure rate of NIV remains substantial, reaching up to 50% [8]. Notably, patients experiencing NIV failure face a staggering 96‐fold higher risk of in‐hospital mortality compared to successful patients [9], with actual mortality rates soaring to 72.7% in some cases [10]. Additionally, patients transitioning to invasive mechanical ventilation after NIV failure exhibit significantly elevated mortality rates [11]. The delayed intubation may exacerbate the condition, further leading to increased mortality rates [12, 13].

Currently, the literature suggests two main methods for determining the effectiveness of NIV: the single‐variable method and the HACOR score. The reported evaluation of the univariate method is only moderately effective [14, 15, 16, 17]. The HACOR score, on the other hand, incorporates five indicators (heart rate [HR], respiratory rate [RR], state of consciousness, pH, and oxygenation index [P/F] ratio) to establish an NIV effectiveness evaluation scale [18, 19]. However, the HACOR score may not fully capture the pathological changes observed in patients with acute hypoxic respiratory failure (AHRF), such as the driving effect of high respiration [20]. Particularly, studies have shown that high tidal volume (VT), rather than RR alone, is independently associated with NIV failure [21, 22]. Addressing the challenge of evaluating respiratory drive changes in patients with AHRF is crucial to improve NIV treatment outcomes, although the latest guidelines on the use of NIV during AHRF have no recommendations [4].

The Volume OXygenation (VOX) index, initially developed to predict treatment failure of high flow nasal cannula (HFNC) therapy, has demonstrated the ability to estimate early increases in respiratory drive. Within the first 2 h, the VOX index exhibits a discriminative potential of 0.88 (95% CI 0.79–0.97) in predicting HFNC failure [23]. Based on this premise, we hypothesize that the VOX index may also serve as a predictive tool for NIV treatment failure and, therefore, undertake retrospective studies to validate its utility.

## 2 Materials and Methods

### 2.1 Subjects

Data were sourced from a prospective multicenter observational study. The original study protocol was approved by the Ethics Committee of the First Affiliated Hospital of Chongqing Medical University (approval number: 2016150). AHRF patients admitted to the intensive care units (ICUs) of three hospitals from September 2017 to September 2021 were included in this retrospective study. Out of 477 patients initially selected for early NIV treatment, 89 patients were excluded due to NIV intolerance, chronic obstructive pulmonary disease (COPD), moderate to severe cardiac failure and data loss, resulting in a total of 388 patients included in the study. As the retrospective design, the need for informed consent was waived and approved by the Ethics Committee of Dazhou Central Hospital (No. 2023060).

The treatment plans for patients were identical, following NIV guidelines. Patients unable to tolerate NIV were treated with HFNC and excluded from the study. Successful NIV patients were transitional to HFNC treatment, while NIV failures were intubated with invasive mechanical ventilation, analgesia, and sedation [24]. The concept of NIV successfully de‐escalation was the case that patients with NIV can be downgraded to HFNC, maintain stable vital signs and arterial blood gas results, and persist for more than 1 day. Those who refused tracheal intubation received palliative NIV. The NIV use and management were built on NIV guidelines, concerning relevant research reports [23, 25]. Noninvasive pressure support ventilation (PSV) was initiated, with an appropriate level of inspired positive airway pressure carefully adjusted to ensure attainment of a target tidal volume exceeding 300 mL (or surpassing 5 mL/kg of predicted body weight), while maintaining a RR below 25 times/min. Improvement in respiratory status and arterial blood gas results after NIV were identified as NIV success. Additional treatment measures included expectorant therapy, anti‐asthmatic medication, anti‐infection treatment, bronchoscopy, nutritional support, early rehabilitation, and intensive care. The SpO_2_, FiO_2_, and VTm (average of three consecutive VT monitoring values/predicted body weight, VT was measured using Philips V60 noninvasive ventilator.) were recorded after 2 h, and the VOX index was computed using the formula: VOX index = SpO_2_/(FiO_2_ * VTm) [23]. The baseline data such as basic information, underlying disease, major diagnosis, Acute Physiological and Chronic Health Score (APACHE II), Glasgow Coma Score (GCS), Sequential Organ Failure Assessment (SOFA) score, and the vital signs and laboratory test results before using noninvasive ventilation were collected. The arterial blood gas values and NIV results at 2 h, 12 h, and 24 h after initiation were collected for all patients. NIV outcomes, intubation status, NIV duration, hospital stay, and mortality rates were registered.

Main outcome measures: endotracheal intubation.

Secondary outcome measures: the status of NIV and arterial blood gas between two groups from initiation to 24 h of NIV, NIV outcomes, NIV time, hospital stay and mortality.

Tracheal intubation criteria [4]: loss of consciousness occurred, with the GCS score being less than 12,cardiac arrest, malignant arrhythmia, or severe hemodynamic instability.

Exacerbation of respiratory failure: At least two of the following conditions must be met: failure to achieve target oxygenation despite high‐concentration oxygen therapy (SpO_2_ < 90% or PaO_2_ ≤ 60 mmHg); respiratory rate exceeding 40 times/min or signs of respiratory distress; respiratory acidosis (pH < 7.25) with progressive hypercapnia; inability to effectively clear a large amount of airway secretions using conventional methods.

### 2.2 Inclusion and Exclusion Criteria [18, 19]

Inclusion criteria: age not less than 16 years old; AHRF defined by PaO_2_/FiO_2_ < 300 mmHg and PaCO_2_ < 50 mmHg at enrollment; RR > 25 beats/min or increased respiratory work; initiation of NIV treatment after the occurrence of AHRF; NIV treatment duration of at least 2 h.

Exclusion criteria: age less than 16 years old; patients with COPD; patients who meet tracheal intubation criteria but refuse to undergo tracheal intubation and opt for NIV as a replacement therapy; patients who received NIV after accidental extubation; patients who underwent preventive NIV following scheduled extubation; patients with moderate to severe cardiac failure New York Heart Association class > II or left ventricular ejection fraction < 50%, and hypoxemia due to heart failure.

### 2.3 Statistical Analyses

Statistical analyses were carried out using SPSS 22.0 statistical software. Measurement data were evaluated using *t* tests or rank sum tests and presented as means ± standard deviations. Categorical variables were analyzed using chi‐square tests. Multivariate logistic regression was used to identify independent risk factors associated with tracheal intubation and death. A significance level of *p* < 0.05 was considered statistically significant. The receiver operating characteristic of tracheal intubation with 2‐h NIV VOX value, the Youden index was 20.45, the VOX value ≤ 20.45 was high risk, and the VOX value > 20.45 was low risk.

## 3 Results

### 3.1 Baseline Data of Enrolled Patients

**FIGURE 1:**
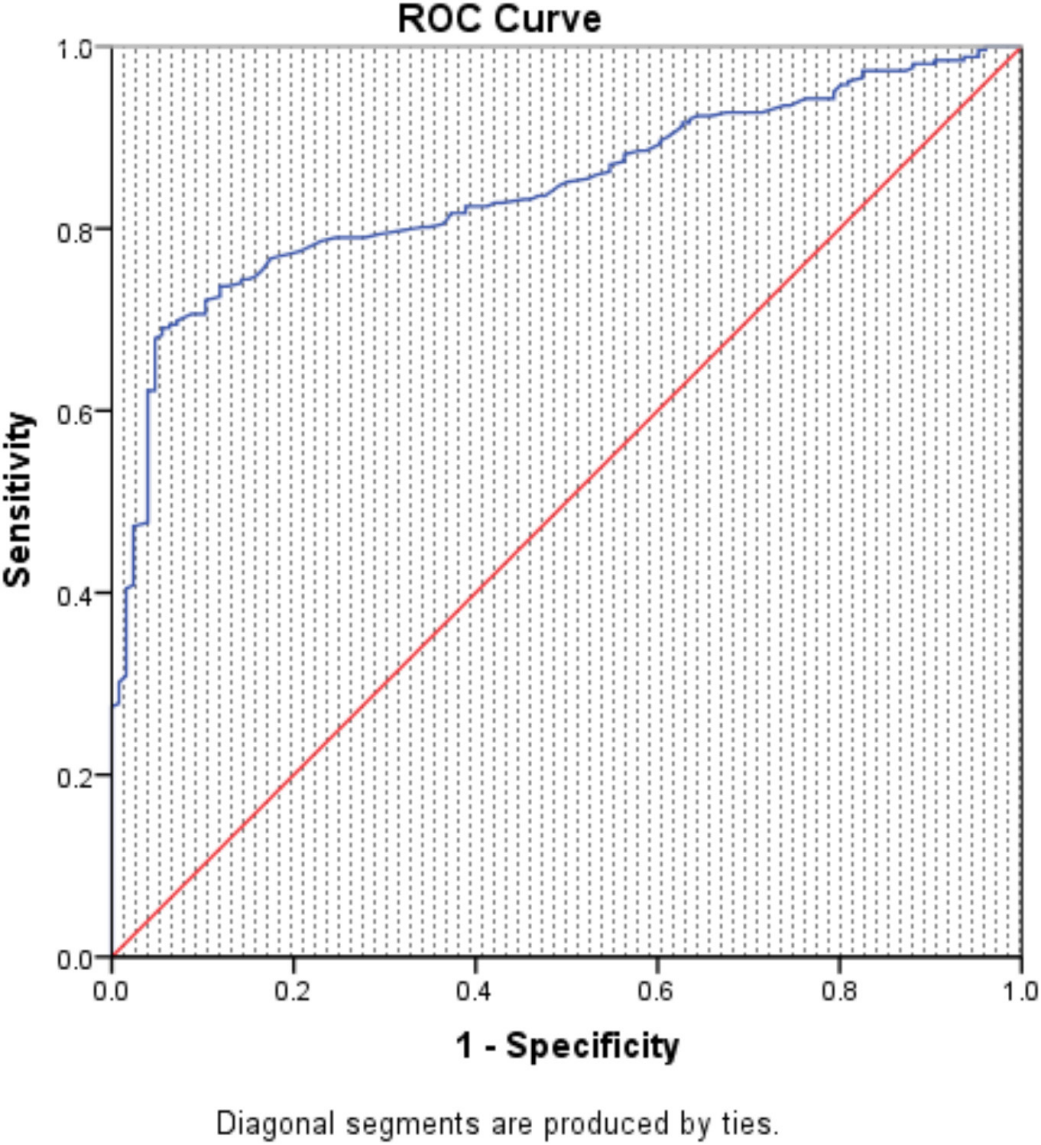
Receiver operating characteristic of tracheal intubation with 2‐h NIV VOX index.

**FIGURE 2:**
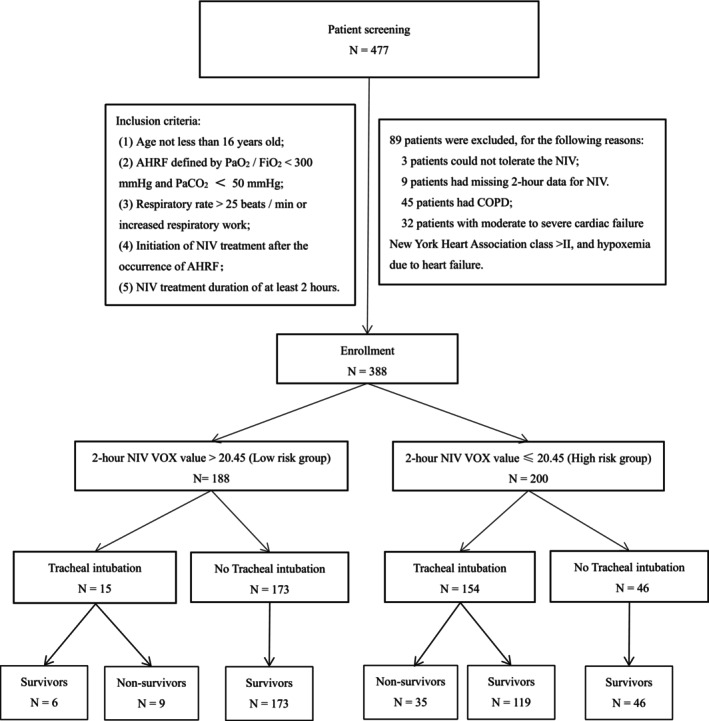
Patient‐screening flowchart.

At 2 h of NIV, the area under the receiver operating characteristic curve for predicting NIV failure evaluated by the VOX index was 0.843 (95% CI 0.805–0.882) (Figure 1). Utilizing a VOX value threshold of 20.45 to predict NIV failure, the sensitivity was 69.1%, and the specificity was 94.4%. Based on the 2‐h NIV VOX Youden index, patients were categorized into the low‐risk group (*n* = 188) with VOX value > 20.45 and the high‐risk group (*n* = 200) with VOX value ≤ 20.45 (Figure 2). Upon enrollment, the high‐risk group exhibited higher SOFA scores, RR, and HR, whereas the low‐risk group had higher P/F ratios (*p* < 0.05). No statistically significant differences were found in other baseline data (Table 1).

**TABLE 1:** Baseline data.

	Total (*n* = 388)	Low‐risk group (*n* = 188)	High‐risk group (*n* = 200)	Difference (95% CI)	*p* value
Age, mean ± SD, years	62 ± 16.8	64 ± 16.9	61 ± 16.6	3.1 (−0.29, 6.42)	0.073
Female, *n* (%)	135 (34.79)	71 (37.77)	64 (32)	—	0.233
Male, *n* (%)	253 (65.21)	117 (62.23)	136 (68)	—	0.233
Underlying disease					
Hypertension, *n* (%)	158 (40.72)	84 (44.68)	74 (37)	—	0.124
Diabetes, *n* (%)	75 (19.33)	40 (21.28)	35 (17.5)	—	0.346
Coronary heart disease, *n* (%)	42 (10.82)	23 (12.23)	19 (9.5)	—	0.386
AIDS, *n* (%)	7 (1.8)	4 (2.13)	3 (1.5)	—	0.642
Malignancy, *n* (%)	51 (13.14)	24 (12.77)	27 (13.5)	—	0.831
Chronic renal insufficiency, *n* (%)	59 (15.21)	33 (17.55)	26 (13)	—	0.212
Chronic hepatic insufficiency, *n* (%)	20 (5.15)	8 (4.26)	12 (6)	—	0.437
Chronic cardiac insufficiency, *n* (%)	139 (35.82)	72 (38.3)	67 (33.5)	—	0.325
Major diagnosis					
Pneumonia, *n* (%)	351 (90.46)	169 (89.9)	182 (91)	—	0.711
Sepsis, *n* (%)	31 (7.99)	14 (7.45)	17 (8.5)	—	0.702
Pancreatitis, *n* (%)	6 (1.55)	5 (2.65)	1 (0.5)	—	0.085
The time of admission					
APACHE II, mean ± SD, score	17 ± 6.6	17 ± 6.3	18 ± 6.9	−1.5 (−2.83, −0.21)	0.735
GCS, mean ± SD, score	14 ± 2	14 ± 1.7	14 ± 2.2	0 (−0.39, 0.38)	0.970
SOFA, mean ± SD, score	6 ± 2.6	5 ± 2.3	6 ± 2.8	−0.8 (−1.35, −0.34)	0.001
Before NIV					
Body temperature, mean ± SD,°C	37.1 ± 0.85	37.1 ± 0.81	37.2 ± 0.88	−0.1 (−0.29, 0.05)	0.167
Respiratory rate, mean ± SD, breaths/min	29 ± 7.1	28 ± 5.6	30 ± 8.1	−2.3 (−3.67, −0.86)	0.002
Heart rate, mean ± SD, beats/min	109 ± 23.5	106 ± 22.8	112 ± 24	−5.2 (−9.91, −0.56)	0.028
SBP, mean ± SD, mm Hg	131 ± 21.5	130 ± 24	132 ± 26.8	−1.6 (−6.72, 3.48)	0.533
DBP, mean ± SD, mm Hg	78 ± 15.9	78 ± 15.2	78 ± 16.5	−0.5 (−3.69, 2.68)	0.755
WBC, mean ± SD, 10^9^/L	11.7 ± 7.96	11.4 ± 6.23	11.9 ± 9.31	−0.5 (−2.06, 1.31)	0.569
PLT, mean ± SD, 10^9^/L	174.8 ± 108.62	187.5 ± 110.49	162.91 ± 105.72	24.7 (3.11, 46.38)	0.187
Hb, mean ± SD, g/L	110.7 ± 31.35	107.3 ± 28.04	113.8 ± 33.94	−6.5 (−12.72, −0.21)	0.132
pH, mean ± SD	7.43 ± 0.09	7.43 ± 0.08	7.43 ± 0.1	0 (−0.02, 0.02)	0.753
PaCO_2_, mean ± SD, mm Hg	33.9 ± 7.52	34.7 ± 7.3	33 ± 7.64	1.7 (0.18, 3.18)	0.455
P/F, mean ± SD, mm Hg	129.9 ± 38.32	139 ± 37.78	121.3 ± 36.92	17.7 (10.19, 25.14)	0.000

Abbreviations: AIDS, acquired immune deficiency syndrome; APACHE II, Acute Physiology and Chronic Health Evaluation II; CI, confidence interval; DBP, diastolic blood pressure; GCS, Glasgow Coma Scale; Hb, hemoglobin; PaCO₂, partial pressure of carbon dioxide; P/F, arterial blood oxygen partial pressure/inhaled oxygen concentration; PLT, platelet count; SBP, systolic blood pressure; SOFA, Sequential Organ Failure Assessment; WBC, white blood cell count.

### 3.2 NIV Status and Arterial Blood Gas Between the Two Groups From Initiation to 24 h of NIV

**FIGURE 3:**
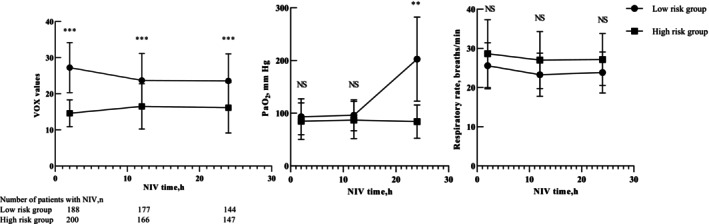
Time course of VOX, RR, and PaO_2_ changes in the two groups.

After 2 h of NIV, both groups showed no difference in NIV success rate and intubation rate after NIV failure. However, after 12 h of NIV, the low‐risk group exhibited a higher NIV success rate (3.72% vs. 0%, *p* < 0.05) and a lower NIV intubation failure rate (2.13% vs. 17%, *p* < 0.05). Similarly, after 24 h of NIV, the low‐risk group demonstrated a higher NIV success rate (21.28% vs. 0%, *p* < 0.05) and a lower NIV intubation failure rate (2.13% vs. 26.5%, *p* < 0.05). The low‐risk group showed a lower VT and MV (minute ventilation volume) at 2 h, 12 h, and 24 h of NIV (*p* < 0.05). Compared with baseline values, both groups exhibited no statistically significant difference in pH and PaCO_2_ after NIV treatment. However, there was a major increase in the P/F ratio after treatment in both groups (*p* < 0.05). Furthermore, the low‐risk group showed a more significant increase in P/F ratio at 2 h, 12 h, and 24 h of NIV (*p* < 0.05), while no statistically significant differences were observed in pH and PaCO_2_ between the two groups (Table 2). By monitoring the temporal changes in VOX, RR, and PaO_2_ in patients, we observed that the VOX values at 2 h, 12 h, and 24 h, as well as the PaO_2_ value at 24 h, were significantly higher in the low‐risk group compared to the high‐risk group (Figure 3).

**TABLE 2:** The status of NIV and arterial blood gas between two groups from initiation to 24 h of NIV.

	Low‐risk group (*n* = 188)	High‐risk group (*n* = 200)	Difference (95% CI)	*p* value
Before NIV	**(*n* = 188)**	**(*n* = 200)**		
pH, mean ± SD	7.43 ± 0.08	7.43 ± 0.1	0 (−0.02, 0.02)	0.753
PaCO_2_, mean ± SD, mm Hg	34.7 ± 7.3	33 ± 7.64	1.7 (0.18, 3.18)	0.455
P/F, mean ± SD, mm Hg	139 ± 37.78	121.3 ± 36.92	17.7 (10.19, 25.14)	0.000
2 h of NIV	**(*n* = 188)**	**(*n* = 200)**		
Successfully de‐escalated, *n* (%)	0 (0)	0 (0)	—	—
Intubation, *n* (%)	0 (0)	0 (0)	—	—
Continuing NIV, *n* (%)	188 (100)	200 (100)	—	—
IPAP, mean ± SD, cm H_2_O	12.3 ± 2.96	12.8 ± 3.02	−0.5 (−1.07, −0.30)	0.404
EPAP, mean ± SD, cm H_2_O	5.5 ± 1.43	5.6 ± 1.56	−0.1 (−0.41, −0.19)	0.618
VT, mean ± SD, mL	459 ± 103.81	529 ± 137.19	−70.0 (−94.36, −45.56)	0.002
MV, mean ± SD, L/min	11.5 ± 3.53	15.1 ± 6.71	−3.6 (−4.72, −2.56)	0.000
pH, mean ± SD	7.44 ± 0.06	7.43 ± 0.08	0 (−0.00, 0.02)	0.157
PaCO_2_, mean ± SD, mm Hg	35.1 ± 7.35	33.5 ± 7.95	1.6 (0.08, 3.14)	0.621
P/F, mean ± SD, mm Hg	182.5 ± 66.14*	144.1 ± 63.6*	38.4 (25.46, 51.36)	0.000
VOX values, mean ± SD	27.3 ± 6.95	14.6 ± 3.73	12.7 (11.53, 13.78)	0.000
12 h of NIV	**(*n* = 177)**	**(*n* = 166)**		
Successfully de‐escalated, *n* (%)	7 (3.72)	0 (0)	—	0.006
Intubation, *n* (%)	4 (2.13)	34 (17)	—	0.000
Continuing NIV, *n* (%)	177 (94.15)	166 (83)	—	0.001
IPAP, mean ± SD, cm H_2_O	12.5 ± 2.92	13.2 ± 3.08	−0.7 (−1.30, −0.03)	0.429
EPAP, mean ± SD, cm H_2_O	5.6 ± 1.42	5.9 ± 1.57	−0.3 (−0.59, 0.05)	0.976
VT, mean ± SD, mL	459 ± 98.98	527 ± 124.97	−68.5 (−92.36, −44.62)	0.017
MV, mean ± SD, L/min	10.6 ± 3.21	14.1 ± 5.24	−3.4 (−4.37, −2.53)	0.000
pH, mean ± SD	7.45 ± 0.05	7.45 ± 0.07	0 (−0.04, 0.13)	0.289
PaCO_2_, mean ± SD, mm Hg	35.5 ± 6.57	33.9 ± 8.09	1.8 (0.19, 3.4)	0.155
P/F, mean ± SD, mm Hg	205.5 ± 75.25*	149.9 ± 69.61*	55 (39.57, 70.47)	0.000
VOX values, mean ± SD	23.7 ± 7.44	16.5 ± 6.26	7.2 (5.76, 8.67)	0.000
24 h of NIV	**(*n* = 144)**	**(*n* = 147)**		
Successfully de‐escalated, *n* (%)	40 (21.28)	0 (0)	—	0.000
Intubation, *n* (%)	4 (2.13)	53 (26.5)	—	0.000
Continuing NIV, *n* (%)	144 (76.6)	147 (73.5)	—	0.482
IPAP, mean ± SD, cm H_2_O	12.4 ± 3.01	13.6 ± 3.02	−1.2 (−1.87, −0.48)	0.946
EPAP, mean ± SD, cm H_2_O	5.6 ± 1.41	6.3 ± 1.72	−0.63 (−0.99, −0.26)	0.182
VT, mean ± SD, mL	462 ± 95.03	541 ± 142.32	−79.8 (−107.77, −51.80)	0.001
MV, mean ± SD, L/min	10.9 ± 3.08	14.8 ± 5.77	−3.9 (−4.95, −2.81)	0.001
pH, mean ± SD	7.46 ± 0.05	7.45 ± 0.06	0 (−0.01, 0.02)	0.437
PaCO_2_, mean ± SD, mm Hg	35.6 ± 6.9	34.2 ± 7.24	1.5 (−0.17, 3.09)	0.080
P/F, mean ± SD, mm Hg	202.8 ± 79.53*	142 ± 62.54*	60.8 (44.29, 77.27)	0.000
VOX values, mean ± SD	23.6 ± 7.47	16.2 ± 7.00	7.4 (5.76, 9.11)	0.000

Abbreviations: CI, confidence interval; EPAP, expiratory positive airway pressure; IPAP, inspired positive airway pressure; MV, minute ventilation volume; NIV, non‐invasive ventilation; P/F, arterial blood oxygen partial pressure/inhaled oxygen concentration; PaCO₂, partial pressure of carbon dioxide; VOX, Volume OXygenation; VT, tidal volume.

* *p* < 0.05 is the comparison before and after NIV treatment.

### 3.3 Outcomes

After treatment, the low‐risk group demonstrated a higher NIV success rate (84.04% vs. 24.5%, *p* < 0.05) and a longer hospital stay ([17.2 ± 12.41] vs. [14.5 ± 12.5] days, *p* < 0.05) compared to the high‐risk group. Additionally, the low‐risk group had a lower tracheal intubation rate (7.98% vs. 77%, *p* < 0.05) and mortality rate (4.79% vs. 17.5%, *p* < 0.05) than the high‐risk group. No statistically significant differences were found between the two groups regarding the number of patients discharged with NIV from the hospital, the number of family‐administered NIV cases, and the duration of NIV treatment (Table 3).

**TABLE 3:** Outcomes.

	Total (*n* = 388)	Low‐risk group (*n* = 188)	High‐risk group (*n* = 200)	Difference (95% CI)	*p* value
NIV outcomes					
NIV successfully de‐escalated, *n* (%)	207 (53.35)	158 (84.04)	49 (24.5)	—	0.000
Endotracheal intubation, *n* (%)	169 (43.56)	15 (7.98)	154 (77)	—	0.000
Discharge with NIV, *n* (%)	55 (14.18)	24 (11.67)	31 (15.5)	—	0.440
NIV time, mean ± SD, days	4.2 ± 4.66	4.2 ± 4.48	4.2 ± 4.85	−0.1 (−0.98, 0.89)	0.125
Hospital stay, mean ± SD, days	15.8 ± 12.51	17.2 ± 12.41	14.5 ± 12.5	2.9 (0.46, 5.37)	0.002
Mortality, *n* (%)	44 (11.34)	9 (4.79)	35 (17.5)	—	0.000

Abbreviations: CI, confidence interval; NIV, noninvasive ventilation.

### 3.4 Independent Risk Factors Associated With Tracheal Intubation and Death

Logistic regression analysis of independent risk factors for tracheal intubation showed that a 2‐h NIV VOX value ≤ 20.45 and APACHE II score were risk factors, while systolic blood pressure and chronic cardiac insufficiency were protective factors against tracheal intubation. Furthermore, multifaceted logistic regression analysis of independent risk factors for death identified a 2‐h NIV VOX value ≤ 20.45 and age as risk factors (Table 4).

**TABLE 4:** Independent risk factors associated with tracheal intubation and death identified by multivariate logistic regression analysis.

Risk factors	OR (95% CI)	*p* value
Endotracheal intubation		
2‐h NIV VOX value ≤ 20.45	51.942 (21.132, 127.672)	0.000
APACHE II, score	1.068 (1.021, 1.117)	0.004
DBP, mm Hg	0.977 (0.959, 0.995)	0.011
Chronic cardiac insufficiency	0.429 (0.233, 0.790)	0.007
Death		
2‐h NIV VOX value ≤ 20.45	4.815 (2.090, 11.091)	0.000
Age, years	1.039 (1.012, 1.066)	0.004

Abbreviations: APACHE II, Acute Physiology and Chronic Health Evaluation II; CI, confidence interval; DBP, diastolic blood pressure; NIV, non‐invasive ventilation; OR, odds ratio; VOX, Volume OXygenation.

## 4 Discussion

VOX index, a new early predictor of HFNC failure in AHRF patients, which estimates early increases in respiratory drive. The VOX index is independently related to a lower risk of HFNC failure within 2 to 6 h of treatment [23]. In the current study, we validated the efficacy of the VOX index in the prediction of NIV failure. The AUC was 0.843, showing a good predictive power of NIV failure.

The results of the retrospective study reported that the low‐risk group, with a VOX value > 20.45, had a significantly higher success rate of NIV and lower rates of tracheal intubation and mortality compared to the high‐risk group. This finding aligns with previous research, demonstrating the effectiveness of the VOX index in predicting NIV failure in patients with AHRF. A comparative analysis of NIV parameter adjustments and arterial blood gas outcomes within 24 h post‐initiation revealed significant differences between groups. The low‐risk group demonstrated lower VT and MV, coupled with higher partial pressure of oxygen to fraction of inspired P/F and VOX values. These findings suggest a more favorable therapeutic response to NIV in the low‐risk group, potentially attributed to reduced respiratory effort, diminished ventilatory demands, and improved patient‐ventilator synchrony resulting from milder respiratory distress. This physiological profile indicates more efficient lung recruitment and ventilator support optimization in patients with less severe disease burden. High respiratory drive in patients with hypoxia can lead to increased VT, contributing to ventilator‐induced lung injury [7, 26, 27], and higher VT, rather than RR alone, has been linked to NIV failure in these patients [21, 22].

Meanwhile, the study outcomes found that the average NIV time between the two groups was roughly equal, because the low‐risk group had a higher success rate of NIV and a larger number of patients as early NIV evacuees, while the high‐risk group had a higher failure rate of early NIV. The hospitalization time of the low‐risk group patients was higher than that of the high‐risk group, due to the low‐risk group having a higher success rate of NIV and a larger number of patients receiving late hospitalization treatment, while high‐risk group having a lower success rate of NIV and the number of patients who received late hospitalization was smaller. Low VOX values may be associated with higher and faster mortality, that is, low VOX values may predict early death. The study outcomes also found that the high‐risk group had a high failure rate of NIV, a high rate of tracheal intubation, and a high mortality rate, which was consistent with pertinent research reports. NIV used in patients with AHRF can improve oxygenation, promote ventilation, reduce respiratory work and dyspnea, avoid intubation, and reduce complications related to invasive mechanical ventilation [28], but patients with NIV failure may have a delay in invasive mechanical ventilation of Tracheal intubation, leading to an increase in mortality [29, 30, 31].

The study also found that the 2‐h VOX value ≤ 20.45 was a risk factor for tracheal intubation and death. This confirms the value of NIV as a therapeutic option for patients with acute left heart failure [7, 8], and the 2‐h VOX value ≤ 20.45 can be used as a predictor of tracheal intubation and death risk.

However, the study has some limitations. It is a retrospective study. During VOX index measurement, NIV parameters were not uniformly standardized across patients. Specifically, PSV pressure levels adjusted to achieve the ventilation target demonstrated significant variability among enrolled subjects. Additionally, the total sample size of the study centers was relatively small, and large‐scale, multicenter randomized controlled trials were absent.

## 5 Conclusions

In conclusion, the VOX index shows promise to serve as an effective evaluation index to predict early NIV efficacy in patients with AHRF, and should be studied in a prospective design to validate these findings. A VOX value > 20.45 after 2 h of NIV treatment can better predict improvements in hypoxia, respiratory drive, and NIV outcomes, guide early tracheal intubation in cases of NIV failure, and have a certain predictive effect on patient outcomes.

## Author Contributions

Xiaoyi Liu conceived this study and is in charge of its completeness. Hui Liu, Lijuan Chen, Hui Ran, Lili Chen, and Xiangde Zheng were involved in research design, research management, data collection, data analysis, and manuscript preparation. Yufeng Wang was involved in research design, data analysis, and manuscript preparation. All authors have been instrumental in the content of the manuscript and approved the manuscript version.

## Ethics Statement

Data were sourced from a prospective multicenter observational study. The original study protocol was approved by the Ethics Committee of the First Affiliated Hospital of Chongqing Medical University (approval number: 2016150). As the retrospective design, the need for informed consent was waived and approved by the Ethics Committee of Dazhou Central Hospital (No. 2023060).

## Conflicts of Interest

The authors declare no conflicts of interest.

## Data Availability

The data that support the findings of this study are available from the corresponding author upon reasonable request. The data are not publicly available due to privacy or ethical restrictions.
